# Hyaluronic Acid Decorated Naringenin Nanoparticles: Appraisal of Chemopreventive and Curative Potential for Lung Cancer

**DOI:** 10.3390/pharmaceutics10010033

**Published:** 2018-03-12

**Authors:** Poonam Parashar, Meena Rathor, Monika Dwivedi, Shubhini A. Saraf

**Affiliations:** Department of Pharmaceutical Sciences, Babasaheb Bhimrao Ambedkar University, Lucknow 226025, India; poonamparashar79@gmail.com (P.P.); meenarathor22.mr@gmail.com (M.R.); monika.nbri@gmail.com (M.D.)

**Keywords:** NSCLC, cell cycle arrest, layer by layer technique, urethane, PCL

## Abstract

Lung carcinoma is the most common cancer in men and second in women (preceded by breast cancer) worldwide. Around 1 in 10 of all cancers diagnosed in men, lung cancer contributed to a total fraction of 20% cancer deaths. Naringenin (NAR) is well known for its chemopreventive properties since ancient times but lacks an appropriate delivery carrier. The objective of present study was to expand the functionality of naringenin loaded poly caprolactone (PCL) nanoparticles in terms of release, chemoprevention and therapeutics. Polymeric nanoparticles such as PCL lack target specificity; hence, surface modification was attempted using layer by layer technique (LBL) to achieve improved and desired delivery as well as target specificity. The designing of Hyaluronic acid (HA) decorated PCL nanoparticles were prepared by utilizing self-assembling LBL technique, where a polycationic layer of a polymer was used as a linker for modification between two polyanionic layers. Additionally, an attempt has been made to strengthen the therapeutic efficacy of PCL nanocarriers by active targeting and overcoming the extracellular matrix associated barriers of tumors using HA targeting cluster determinant 44 receptor (CD44). Cell cytotoxicity study on A549 cells and J774 macrophage cells depicted enhanced anticancer effect of NAR-HA@CH-PCL-NP with safe profile on macrophages. Uptake study on A549 cells advocated enhanced drug uptake by cancer cells. Cell cycle arrest analysis (A549 cell lines) demonstrated the superior cytotoxic effect and active targeting of NAR-HA@CH-PCL-NP. Further chemopreventive treatment with NAR-HA@CH-PCL-NP was found effective in tumor growth inhibitory effect against urethane-induced lung cancer in rat. In conclusion, developed formulation possesses a promising potential as a therapeutic and chemopreventive agent against urethane-induced lung carcinoma in albino wistar rats.

## 1. Introduction

Lung cancer (LC) is the most frequent cancer occurring globally reported in both men and women [1]. It is the second most common cancer accounting for 20% (1.6 million per year) of total cancer mortality [2]. On a broad basis, LC is divided into two major types, non-small cell lung cancer (NSCLC) and small cell lung cancer (SCLC), of which NSCLC alone accounts for 85% [3,4]. Poor prognosis, lack of early detection, development of resistance to chemotherapeutic agents and overall five-year survival rate of <15% are the foremost obstacles for effective LC treatment [5]. Currently, most opted treatment strategies include radiotherapy and chemotherapy which pose challenges to effective treatment of LC [6]. These conventional treatments have discouraging outcomes due to limitations, namely dose-dependent toxicity, low selectivity, resistance development and compromised delivery of chemotherapeutic agents to lung tissue due to high tumor interstitial pressure [7]. Led by these limitations, a major paradigm shift in the management of LC therapy has occurred as innovative researchers aimed to develop newer drugs/delivery systems to specifically target over-expressed factors such as cluster determinant 44 receptor (CD44), EGFR (epidermal growth factor receptor), folate receptor and so on. A wide variety of polymeric nanoparticles (NPs) have been reported which can be surface engineered so as to protect the agent from reticuloendothelial system RES uptake during its circulation in the blood stream and may serve the dual purpose of targeting cancer cells while protecting normal non-targeted tissues from toxicity [3,8]. Previous investigations proved these surface modified NPs to be biocompatible, having better cellular uptake and ability to target tumor tissue [9]. These surface engineered NPs may be an alternative approach for targeted delivery to tumor cells that may improve efficacy and reduce adverse effects of cancer therapy [10]. Among various polymeric NPs, PLGA (Poly lactic glycolic acid) and PCL (poly capro lactone) have attained attention to be formulated as targeted delivery systems. This is due to their biocompatible, biodegradable nature. Vangara et al. proved the potential of Hyaluronic acid(HA) decorated PLGA-(Polyethylene glycol) PEG NPs in targeting tumor tissue over expressed with CD44 [11]. Narsireddy et al. reported gene delivery to LC tissue utilizing PCL NPs [12]. The polymeric NPs have shown great potential in targeting cancer tissues. The cancer cells necessitate additional vitamins and nutrients such as folic acid, biotin, for their active proliferation, thus a quick uptake of NPs carrying such molecules is observed via these overexpressed receptors. When a surface modified NP approaches these overexpressed receptors, they rapidly internalize it and the drug is directly delivered to these cells [1].

The use of HA for targeting tumors has been reported in diverse literature as it possesses number of qualities, viz. biocompatibility and biodegradability owing to its negative charge which can interact better to tumor receptor-like CD44 [13]. Cancer cells showed a higher expression of CD44 level when compared with normal cells and was recognized as a potential therapeutic target in cancer therapy [14]. HA is a type of linear mucopolysaccharide composed of alternately repeated *N*-acetylglucosamine and glucuronic di-saccharide, and it constitutes the main component of the extracellular matrix which helps in better interaction with the biological membranes [15]. HA is regarded as an extracellular matrix component that facilitates cell locomotion and proliferation. HA has often been utilized in surface modification of drug carrier due to its ability to specifically target CD44 receptors to effectively inhibit tumor growth [16]. HA is a major ligand of CD44 and these receptors are over expressed in tumor cells, thus HA can be used for better targeting to cancer cells. Earlier researchers have taken the benefits of HA in designing targeted delivery systems for cancer therapy. Wang et al. reported enhanced drug accumulation in tumor cells and improved antitumor efficiency of chemotherapeutic agent by designing surface modified polymeric NPs utilizing HA [14,17]. Li et al. successfully formulated HA modified zirconium phosphate NPs for lung cancer therapy and found enhanced in vitro efficacy against A549 cell lines as well as improved in vivo anticancer potential having minimized toxic side effects of drug as well as strong tumor-suppression [18]. Tashiro et al. reported successful delivery and stability of HA via peroral route, which makes it a molecule of choice for tumor targeting through naringenin NAR loaded NPs.

Chemoprevention through natural products has alarmed a huge population because of its natural occurrence, no or minimal adverse effect and low price and thus scientists are working on making an effective delivery system and dosage form to be administered by patients [19]. NAR is a flavanone (5,7,4-trihydroxyflavanone or 5,7-dihydroxy-2-(4-hydroxyphenyl)chroman-4-one) found especially in citrus fruits, and grapefruit [20]. NAR has attracted a number of scientists because of its anticancer, antimutagenic, anti-inflammatory, antiatherogenic, antifibrogenic and free radical scavenging properties [21]. NAR is reported to induce cytotoxicity and apoptosis in various cancer cell line and its treatment at a similar dose showed no toxic effect on normal cells [22]. Reported in previous literature, NAR has proven its potential as an immunomodulator for inhibiting lung fibrosis and metastasis [23]. Chang et al. also reported inhibition of matrix metalloproteinases-2 and -9 in LC and preventing its migration utilizing NAR [24]. Previous studies have reported NAR to inhibit both cell proliferation and motility by interfering with the phosphoinositide 3-kinase (PI3K) and Mitogen-activated protein kinase MAPK pathways which results in cell growth inhibition and upregulation of autophagic protein followed by autophagy-mediated cell death. Previous studies have reported NAR’s inhibitory effects on cell proliferation and viability as a result of extracellular signal-regulated kinase ½ (ERK1/2) inhibition. Further, NAR is also known to upregulate the expression of cytochrome P450 1A1 enzyme (CYP1A1), Proliferating cell nuclear antigen (PCNA) and nuclear factor κB (NF-κB), an intracellular reactive oxygen species (ROS) production coupled with the concomitant activation of the caspase cascade signaling pathway. NAR inhibits migration of lung cancer cells via the inhibition of matrix metalloproteinases-2 and -9. NAR has a greater affinity to ERb than to ERa (ER- human oestrogen receptor), thus modulating the 17b-estradiol (E2)-induced gene transcription [24,25,26,27,28].

NAR faces a challenge due to its poor oral bioavailability, low aqueous solubility, limited permeability, instability and extensive first-pass metabolism which limits its use as a drug. The present work envisages overcoming the problems of poor water solubility and oral bioavailability of NAR by preparing surface decorated NAR-PCL nanoparticles. In this study, an attempt has been made to design surface functionalized hyaluronic acid-chitosan-PCL nanoparticles by using layer by layer technique (LBL) for targeted delivery to lung cancer cell. PCL and NAR both have a hydrophobic surface, thus they can be used in the same phase for fabrication of PCL nanoparticles. Moreover, PCL has low degradation rate, it inherited significant potential for sustained release and the enhancement of circulation time of loaded drug is expected [29]. Since, both PCL and HA are polyanionic in nature, chitosan (CH) is used as a polycationic layer for fabrication of the LBL assembly of polyelectrolytes and the mucoadhesive property of CH is also exploited for designing as our study aimed at oral delivery of naringenin, thereby; a sustained and retarded release profile is desired to achieve the purpose. Layer by layer assembly provides a multifunctional carrier system in respect of releasing the payloads in a controlled manner.

Cell cycle study on A549 cells advocates the apoptotic effect of the merit formulation. In vivo anticancer activity was also evaluated in urethane-induced (reported in various studies, viz. Hamzawy et al. and Hori et al [30]. albino wistar rat model. Further ex vivo intestine permeation study supports functionality of HA decorated NPs for efficient oral delivery.

## 2. Materials and Methods

### 2.1. Materials

Naringenin, polymer polycaprolactone (PCL) (Mw = 42,500–65,000), and surface modifier hyaluronic acid (HA) (Mw = 200 kDa) were purchased from M/s Sigma Aldrich, St. Louis, MO, USA. Chitosan, tween 80 and urethane of were purchased from M/s Hi-media chemicals, Mumbai, India. All other chemicals were of analytical grade and purchased from Merck Specialities Pvt. Ltd. Mumbai, India. In-house double distilled water was used throughout the experiment.

### 2.2. Animals

Albino wistar rat (male, seven-week-old) were procured from Council of Scientific & Industrial Research -Indian Institute of Toxicology Research CSIR-IITR, Lucknow, India. They were fed with synthetic pellet diet (M/S Provimi Animal Nutrition India Pvt. Ltd. Bangalore, India) and water ad libitum. Animals were kept in polypropylene cages in a well-ventilated animal house at temperature 22 °C ± 2 °C. All experiments were done in accordance with the guidelines of the Institutional Animal Ethical Committee. Animals were divided into 6 groups having 6 animals each under a 12 h light/dark cycle, and acclimatized for 2 weeks. This study was approved by the Institutional Animal Ethical Committee REG. No. 809/PO/Re/S/03/CPCSEA dated 22 April 2017 BBDNIIT. All animals were handled as per institutional animal ethics norms, and care was taken that all guidelines were followed with a human approach.

#### Cell Lines

Human non-small-cell lung carcinoma (NSCLC) cell lines (A549) were obtained from American Type Culture Collection (ATCC, Rockville, MD, USA). Cells were maintained in appropriate culture medium in Dulbecco’s Modified Eagle’s Medium DMEM supplemented with 10% fetal bovine serum and 1% antibiotic (penicillin) solution. A549 cells were maintained at 37 °C with 5% CO_2_ in incubator [22].

### 2.3. Methods

#### 2.3.1. Preparation of Naringenin Loaded Poly-ε-caprolactone (PCL) Nanoparticles (NAR-PCL-NP)

NAR loaded PCL nanoparticles were prepared by nanoprecipitation method as reported in previous literature [24]. NAR-PCL-NPs were prepared by nanoprecipitation technique, PCL in varying concentration of 1% *w*/*v*, 0.5% *w*/*v* and 0.75% *w*/*v* (10 mL) was taken and drug NAR (5 mg) dissolved in (1 mL) of acetone. The resulting solution was added drop wise to 0.5% *v*/*v* tween-80 (10 mL) with the help of a syringe under continuous stirring over magnetic stirrer (Tarsons-Spinot MC 01, Delhi, India). This solution was stirred overnight to obtain NAR-PCL-NPs. The liquid sample was lyophilized (Christ Alpha 1-4 lyophilizator, Osterodo, Germany) to get dry powdered nanoparticles. The prepared lyophilized NAR-PCL-NPs were stored safely, till further use at room temperature.

#### 2.3.2. Surface Modification of NAR-PCL-NP

Surface modification of NAR-PCL-NP was done utilizing layer by layer method [31,32]. Direct coating of HA over PCL is not possible as both HA and PCL bear negative surface charge, resulting in charge repulsion. Thus, to coat the NAR-PCL-NP with HA, an intermediate layer of chitosan having positive charge was coated in between PCL and HA. For this, NAR-PCL-NP (NAR loaded PCL nanoparticles) were added drop wise into 0.01% *w*/*v* chitosan solution in the ratio of 1:2, with continuous stirring for 2 h (Tarsons-Spinot MC 01, Delhi, India) to get chitosan coated nanoparticles NAR-CH-PCL-NP. The zeta potential of the resulting solution was determined to confirm the coating and the charge reversal using NanoPlus-3, zeta/nano particle analyzer Particulate System Analyzer, Yokohama, Japan.

After charge reversal, the prepared NAR-CH-PCL-NP i.e., chitosan coated nanoparticles were added to 0.01% *w*/*v* of HA solution in the ratio of 1:3 to obtain NAR-HA@CH-PCL-NP. Zeta potential of the prepared sample was determined to identify charge on the surface of NAR-HA@CH-PCL-NP using zeta-sizer and particle size analyzer (NanoPlus-3, Yokohama, Japan). Finally, the prepared surface modified NAR-HA@CH-PCL-NP were obtained and were lyophilized (Christ Alpha 1-4 lyophilizator, Osterodo, Germany) to get powdered nanoparticles. The final formulation was stored at a low temperature of 4 °C till further use.

### 2.4. Characterization of Prepared NAR-HA@PCL-NP

#### 2.4.1. Particle Size Analysis and Surface Charge

The particle size of NAR-HA@CH-PCL-NP was determined using laser light scattering-based particle size analyzer (NanoPlus-3, Yokohama, Japan). Study was performed in triplicate, and average values with standard deviation were reported. The samples were reconstituted in phosphate buffer pH 7.4 prior to analysis. The experiments were done in triplicate.

#### 2.4.2. Transmission Electron Microscopy

Surface morphology of surface modified (NAR-HA@CH-PCL-NP) NAR nanoparticles was examined, through TEM (H-750, Hitachi, Tokyo, Japan). For TEM analysis, samples were prepared by mounting a drop of previously diluted sample over 400 mesh copper grid, followed by drying using IR lamp. The excess sample was removed with filter paper. Sample was then assessed under 100 kV voltage and 2000 magnification over TEM (Hitachi H 7500). After sample preparation, photomicrographs were captured for interpretation of results.

#### 2.4.3. Entrapment Efficiency

Entrapment efficiency was determined by centrifugation method. Formulation (1.5 mL each) was centrifuged (REMI CPR-24) for 15 min at 10,000 rpm at 4 °C to separate free drug. The pellet and supernatant were separated. The supernatant was collected to determine free drug present in it and the concentration of free drug was measured by determining absorbance at 285 nm using UV spectrophotometer (Labtronics LT-2910, Haryana, India). All the experiments were done in triplicate. The entrapment efficiency was calculated using formula:

$$\%\;\mathrm{EE}\;=\;\frac{W\;\mathrm{initial}\;\mathrm{drug}-\mathrm{Wfree}\;\mathrm{drug}\;\times \;100}{W\;\mathrm{initial}\;\mathrm{drug}}$$

#### 2.4.4. In Vitro Dissolution Studies

In-vitro drug release of NAR loaded nanoparticles NAR-PCL-NP, NAR-CH-PCL-NP and NAR-HA@CH-PCL-NP was performed using dialysis membrane (Hi-media, Mumbai, India) having molecular weight cut off range of 12,000–14,000 dalton. Dialysis membrane was activated with phosphate buffer pH 7.4/acidic buffer pH 1.2, prior to experiment. USP dissolution apparatus type-II (Veego, Mumbai, India) was used for in vitro release study. The in vitro release profiles of various batches were performed using two different dissolution media in order to mimic the GI conditions and to determine their stability in gastric fluids of various pH. In vitro release profiling was carried out initially in simulated gastric fluid (SGF) of pH 1.2 (2 h) followed by simulated intestinal fluid (SIF) of pH 7.4 as the release media. The simulated gastric fluid (pH 1.2) comprised of sodium chloride (2.0 g), pepsin (3.2 g) and hydrochloric acid (7.0 mL) with the volume being made up to 1000 mL with water. The simulated intestinal fluid (pH 7.4) contained monobasic potassium phosphate (6.8 g), 0.2 *N* Sodium hydroxide (180 mL) and pancreatin (10.0 g) and volume was made up to 1000 mL with water. Formulation equivalent to 5 mg of NAR was taken and packed in the sac made of a dialysis membrane; the sac was tied to the paddle of the shaft in such a way that membrane just touches the dissolution medium surface. The dissolution medium was stirred at 50 rpm. The release study was carried out for 24 h and an aliquot of 3 mL of the sample was withdrawn from the cylinder at pre-determined time intervals and replenished with fresh medium. The aliquots were filtered through 0.45 mm membrane filters. Drug concentration was determined spectrophotometrically at 285 nm using UV spectrophotometer (Labtronics-2910, Haryana, India). Cumulative percent drug release was plotted as a function of time, to determine the release of NAR from NAR-PCL-NP, NAR-CH-PCL-NP and NAR-HA@CH-PCL-NP nanoparticles. All experiments were conducted in triplicate.

#### 2.4.5. Release Kinetics Modeling

The data were analyzed for various kinetic models viz. zero order, first order, Higuchi model, Korsmeyer-Peppas, etc. The optimized formulation NAR-HA@CH-PCL-NP was subjected to graphical treatment to predict the kinetics of drug release using goodness of data fit approach.

### 2.5. In Vitro Cell Culture Study

#### 2.5.1. Cell Cytotoxicity Study by MTT Assay

Cytotoxicity of NAR was assessed on cancer cells and normal cells by MTT assay to evaluate the anticancer efficacy in vitro and safety on normal cells. Lung cancer cells A549 and J774 macrophages were cultured in 96 well plate with cell density (1 × 10^3^ cells/well) and incubated overnight at 37 °C, 5% CO_2_ in DMEM media comprising 10% FBS. The cells were treated with various concentrations of NAR and NAR-HA@CH-PCL-NP ranging from 0 to 50 μM equivalents NAR. After 48 h of incubation, MTT dye (8 µL, 5 mg/mL) was added for 4 h leading to formation of MTT-formazan crystal, dissolved with Dimethyl sulfoxide DMSO. The optical density was determined via Elisa plate reader at 540 nm.

#### 2.5.2. Cell Uptake Study

Drug internalization in lung cancer cells A549 was assessed by fluorescence activated cell sorter (FACS) instrument (BD Biosciences, FACS Aria, Heidelberg, Germany). The Fluorescein isothiocyanate FITC loaded nanoparticles were developed using same procedure as used for NAR-PCL-NP and NAR-HA@CH-PCL-NP to evaluate the function of delivery system in enhanced uptake of drug in the cancer cells. A549 cells were cultured in a 6 well plate in fresh DMEM media following similar method as described in previous assay and incubated in CO_2_ incubator for 24 h at 37 °C. Later, the cells were treated with FITC-labelled NAR-PCL-NP and NAR-HA@CH-PCL-NP for 4 h. After the end of incubation duration, the cells were washed with PBS thrice and analyzed via flow cytometric analysis at an excitation wavelength of 480 nm and an emission wavelength of 550 nm.

#### 2.5.3. Cell Cycle Arrest by NAR-HA@CH-PCL-NP

Estimation of cell cycle arrest caused by NAR and its nanoformulation has not been reported so far in NSCLC. Henceforth, it becomes obligatory to identify the stage of cell cycle arrest by NAR in NSCLC. With this aim, A549 cells were grown at a density of 1 × 10^6^ cells/well in 6-well plate and exposed with native Control, Blank HA@CH-PCL-NP, NAR and NAR-HA@CH-PCL-NP for 48 h. Propidium iodide method was employed and fluorescence was measured for cell cycle analysis, using flow cytometry (BD FACS Array). For this, the cells were harvested in PBS and fixed in cold 70% ethanol for 30 min at 4 °C. The cells were washed twice using PBS (phosphate buffer saline) followed by centrifugation for 15 min at 850× *g* (REMI CPR-24). The cells were then treated with ribonuclease (50 μL of a 100 μg/mL stock of RNase). To this, finally 200 μL propidium iodide was added and forward scatter, side scatter and histograms were analyzed.

### 2.6. In Vivo Study

#### 2.6.1. Permeation Measurements across Excised Rat Small Intestine [33]

In permeation measurement study, ex vivo absorption of FITC loaded HA modified NPs (FITC-HA@CH-PCL-NP) was investigated to determine drug permeation in excised rat small intestine. For this study, small intestine was isolated from freshly excised rat and washed with Krebs-Ringer buffer (KRB) and each piece was expurgated to 5 cm. Afterwards, the excised tissues were fastened to a suture from one end. FITC loaded HA modified NPs (FITC-HA@CH-PCL-NP) and PCL-NPs (FITC-PCL-NPs) were prepared by pre-loading of FITC (0.5 μg/mL) via nanoprecipitation method. The protocol was performed in dark conditions. FITC- HA@CH-PCL-NPs and FITC-PCL-NPs were put in the free end of the intestinal sac, 0.2 mL of Krebs-Ringer buffer was added, and the other free end was also tied up. These well packed sacs were immersed in 10 mL of oxygenated Krebs-Ringer buffer in vials. The process was assisted with gentle shaking (100 rpm) at 37 °C. From the sample, an aliquot of 0.5 mL was withdrawn from each vial at pre-determined time intervals and replenished with fresh buffer. Samples were evaluated at excitation wavelength of 490 nm and emission wavelength 525 nm using a fluorescence spectrophotometer.

#### 2.6.2. In Vivo Efficacy Study

The protocol for animal testing was approved through REG. No. 809/PO/Re/S/03/CPCSEA. Albino wistar rat both male and female, weighing 120–150 g were used for the experiment. Animals were randomized and divided into six groups of 9 animals each. Group I (negative control); Group II (toxic, Urethane 1 g/kg, i.p.); Group III (NAR, 50 mg/kg), Group IV (receiving NAR-PCL-NP dose: 50 mg/kg, oral.); Group V (NAR-HA@CH-PCL-NP 50 mg/kg, oral.); and group VI (NAR-HA@CH-PCL-NP) as preventive 15 days prior to treatment. Lung cancer was induced by three consecutive i.p. injections of urethane, within a gap of 48 h in a week. The development of lung tumors was initiated over a period of 9–12 weeks. One animal was taken as a representative from each group (except negative control) and sacrificed to confirm lung tumor development at 4, 9 and 12 weeks. The other animals in the group were considered to be LC positive if the sacrificed one was found to be so. The blood samples were collected under chloroform anesthesia through retro orbital plexus in centrifugation tubes for further analysis. The blood samples were incubated at 37 °C for 1 h and centrifuged at 10,000 rpm for 15 min to collect serum. The serum samples were stored at −20 °C till further use. Animals were sacrificed on the 21st and 35th (for preventive group) day after treatment and lung tissues were procured till further evaluation at −80 °C [34,35].

##### Histopathology

Lung tissues from all the five groups were stored in 10% buffered formalin for histopathology. The tissues were usually processed, entrenched with paraffin wax, and sectioned (3–5 μm) via rotary Microtome (YSI-060 Yorco, Ghaziabad, India). Sections thus obtained were unflustered over the glass slide, deparaffinized, and stained with hematoxylin and eosin. The sections of the specimen were observed and photographed at 40× using digital biological microscope (N120, BR-Biochem Life Sciences, New Delhi, India.

##### Biochemical Estimation

The LC tissues (10% *w*/*v*) were homogenized in 0.15 M KCl and centrifuged at 10,000 rpm. The supernatants were scrutinized for biochemical parameters including thiobarbituric acid reactive substances (TBARS), superoxide dismutase (SOD), catalase and glutathione (GSH) using the methods established in our laboratory. For TBARS estimation, suspension medium (1 mL) was taken from the supernatant of the 10% tissue homogenate and centrifuged at 10,000 rpm. Then, 0.5 mL of 30% Trichloro acetic acid (TCA) followed by 0.5 mL of 0.8% Thiobarbituric acid reactive species (TBA) was added to it. The tubes were covered with aluminum foil and kept in a shaking water bath for 30 min at 800 °C. After 30 min, tubes were taken out and kept in ice-cold water for 15 min. They were then centrifuged at 3000 rpm for 15 min. The absorbance of supernatants was recorded at 540 nm at room temperature against appropriate blank. Blank consisted of 1.0 mL distilled water, 0.5 mL of 0.8% TBA solution, and 0.5 mL of 30% TCA solution. All the experiments were done in triplicate and was subjected to statistical analysis using Graph Pad Prism (5.01), San Diego, California software [36].

### 2.7. Storage and pH-Dependent Stability Studies

Stability studies of NAR-HA@CH-PCL-NP were done as per International Conference on Harmonisation ICH guidelines over a period of 3 months. The formulations were evaluated for particle size, zeta potential and drug content. The formulations were kept in transparent containers over a period of 90 days for stability assessment at 4 °C ± 1 °C and 25 °C ± 2 °C. Samples were assessed at 0, 15, 30, 45, and 90 days. All experiments were performed in triplicate. Stability of NAR-HA@CH-PCL-NP was also evaluated at different pH conditions simulating gastrointestinal tract (GIT) viz. pH 2, 6.8 and 7.4. NAR-HA@CH-PCL-NP were incubated at pH 2.0 and simulated gastric fluid for 2 h, at pH 6.8 and simulated intestinal fluid for 6 h, in DMEM pH 7.4 supplemented with 10% FBS (culture medium) for 24 and in PBS pH 7.4 for 7 days. Particle size, Poly dispersity index (PDI) and zeta potential were measured after incubation period.

### 2.8. Factorial Design

A 2^3^ randomized full-factorial design was used in this study. In this design, three prime factors or variables were evaluated, each at two levels. A factorial design evaluating three factors at all combinations for each level would result in a full factorial design consisting of 8 runs. Thus, experimental trials were performed at all eight possible combinations. The amount of PCL (A) and tween 80 (B) were selected as independent variables along with the stirring speed (C) as third variable. The particle size and PDI were the outputs as dependent variables. The design layout is depicted in Table 1.

### 2.9. Statistical Design

Data were expressed as mean and standard deviation of separate experiments (*n* = 3).

A commercially available software program was used (Design Expert, Version 8, Stat-Ease Inc., Minneapolis, MN, USA) for factorial design and related ANOVA. The experimental design chosen was response surface, two-factor, three-level factorial out of which nine formulations were prepared and Graph Pad Prism 5 software was used for rest of all statistical analyses. The significance level was *p* < 0.05 for all the experiments.

## 3. Results

### 3.1. Formulation of HA Decorated NAR-Loaded Nanoparticles and Characterization

Present investigation promises the designing of HA decorated layer by layer assembled PCL NPs sandwiched with CH for oral delivery to attenuate lung cancer.

PCL NPs were optimized using 2^3^ factorial designs, where PCL concentration, tween-80 concentration and varying stirring speed (Supplementary Materials, Table S1) were used as independent variables to form batches. Using these factors, eight pre-optimized formulations of different composition were proposed through Design Expert^®^ software. The factor levels were coded into the amount of ingredients i.e., PCL concentration, Tween 80 concentration and varying stirring speed for nanoparticles are listed in Supplementary Materials, Table S2. An inference was drawn from the factorial design study that variable A and C have significant interactions (*p* < 0.0345) and final equation derived from the interaction of actual factors for particle size was stated as:

Particle size = −1.82575 + 0.16500 × PCL + 3.77150 × Tween 80 + 2.43250E-003 × stirring speed − 1.27800 × PCL × Tween 80.

The study design for second the factor i.e., PDI, variables A and B have significant interactions (*p* < 0.0255) and effect of variable C is not significant. Thereby, final equation for PDI in terms of actual variables was derived as:

PDI = −1.82575 + 0.16500 × PCL + 3.77150 × Tween 80 + 2.43250E-003 × Stirring speed − 1.27800 × PCL × Tween 80.

**Figure 1:**
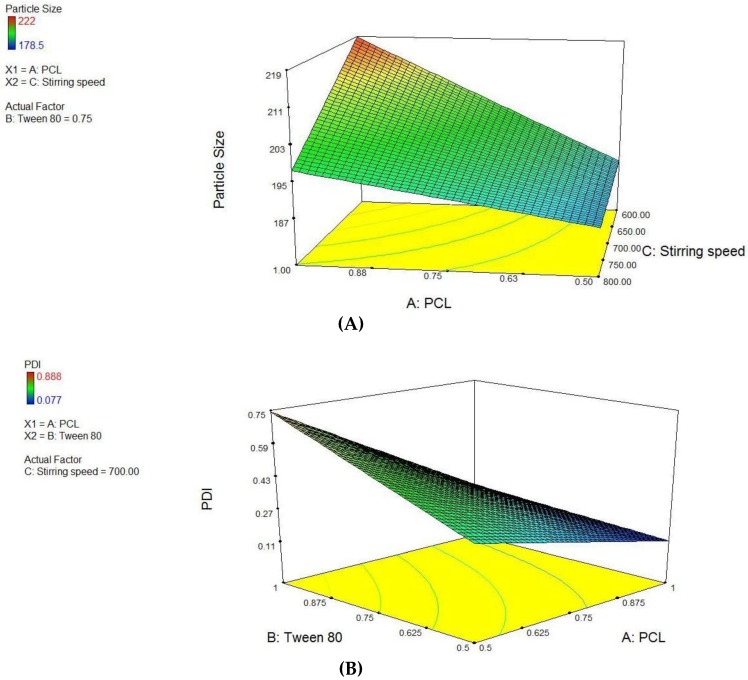
Surface response plot showing influence of: (**A**) PCL and Stirring Speed on Particle size; and (**B**) PCL and Tween 80 on PDI.

**Figure 2:**
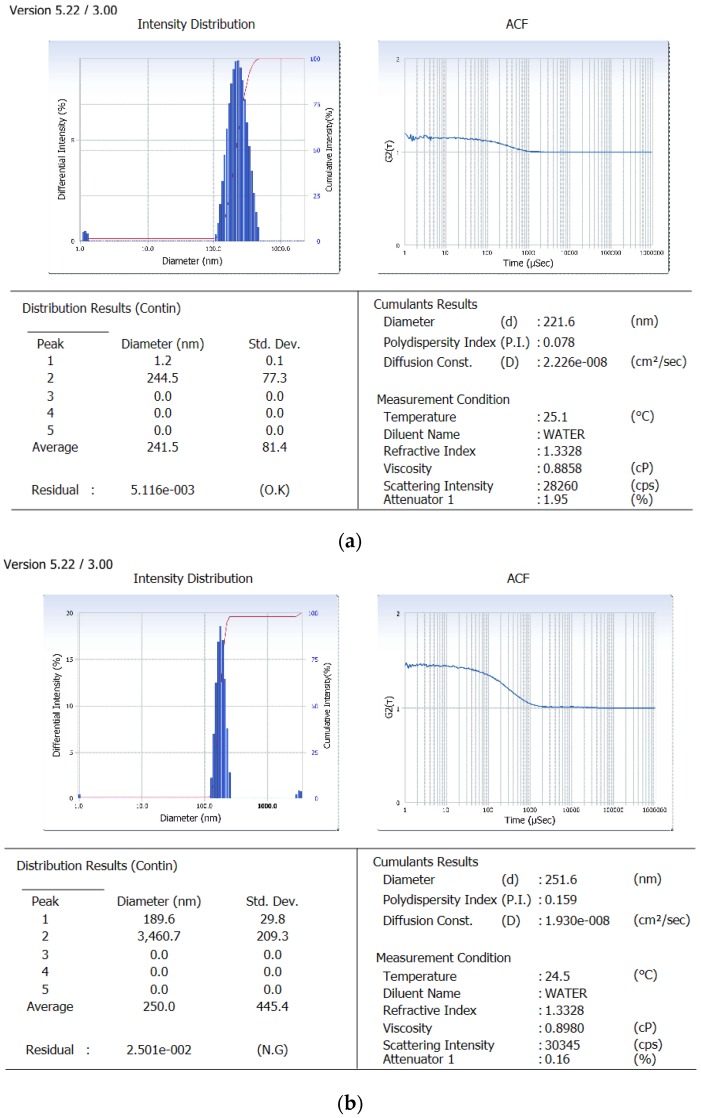
Particle size image of: (**a**) NAR-PCL-NP; and (**b**) NAR-HA@CH-PCL-NP.

Derived from the theoretical prediction F2 with the coded output of variables (A = +1, B = −1 and C = 1) with maximum desirability was correlated with characterization parameters such as particle size, zeta potential percent drug loading and entrapment efficiency (recorded in Table 1). The actual outputs compelled to rely on the theoretical results and F2 with particle size 222 ± 0.36 nm, zeta potential −19.54 ± 0.015, PDI = 0.077 ± 0.0011, % drug loading 3.92 ± 0.012 and % entrapment efficacy = 75.8 ± 0.23 was selected as optimized formulation NAR-PCL-NP for further surface modification. The surface response plot, i.e., area of desired spaces (dark area), was specified by putting some constrains to the dependent variables and an optimized formulation (shown in Figure 1) was prepared taking particle size. The surface modification was done by CH preceded by HA on the outer surface for targeting of CD44 receptors. The particle size of the CH coated PCL-NPs (NAR-CH-PCL-NP) and HA decorated NAR-HA@CH-PCL-NP was found to be 221.6 ± 1.21 nm and 251.6 ± 3.22 nm with a good polydispersity index of 0.078 and 0.263 indicating that the formulation was homogenous with respect to particle size (Figure 2). It was perceived from the surface response plot that as the PCL concentration increases the particle size increases. Although there was an insignificant effect of PCL concentration on PDI, tween 80 was found to play a significant role in PDI, and as the concentration of tween 80 increases, the PDI increases.

**Figure 3:**
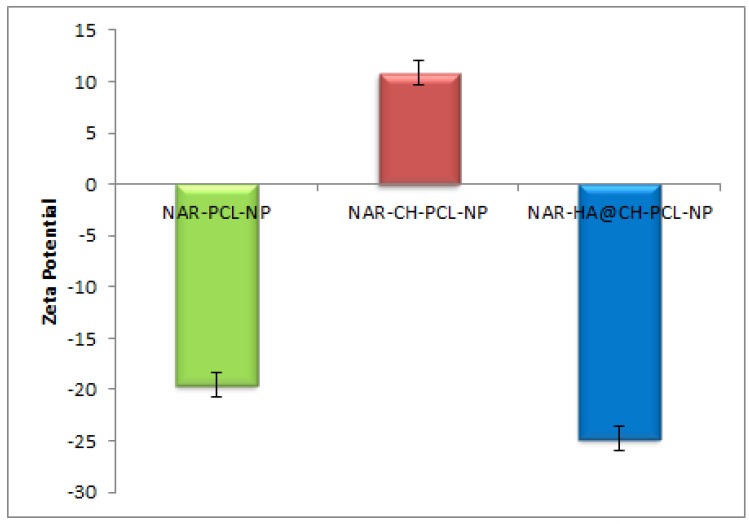
Zeta potential of NAR-PCL-NP, NAR-CH-PCL-NP and NAR-HA@CH-PCL-NP.

Dual surface modification by LBL technique was confirmed by the charge developed by the polymer used for the surface coating. Thereafter, the zeta potential of the layer by layer assembly system was measured at each step during fabrication of modified nanoparticles. Zeta-potential of NAR-PCL-NP was found to be −19.54 ± 0.56 mV due to the negative charge of PCL. The modification was confirmed by the charge reversal of the NAR-CH-PCL-NP from −19.54 mV ± 0.89 to +10.8 ± 0.67 mV due to chitosan coating as shown in Figure 3. Being cationic in nature, at final step HA decoration reverses charges to negative side, i.e., from +10.8 ± 0.39 mV to −24.24 ± 0.015 mV. The threshold value for zeta potential is often considered to be ±20 mV for particles to be stable, although it is not always the case and stable NPs of much lesser zeta potential have been reported. In the present study, stable surface decorated NAR-HA@CH-PCL-NP were developed and further subjected to surface morphology evaluation.

### 3.2. Shape and Surface Morphology

**Figure 4:**
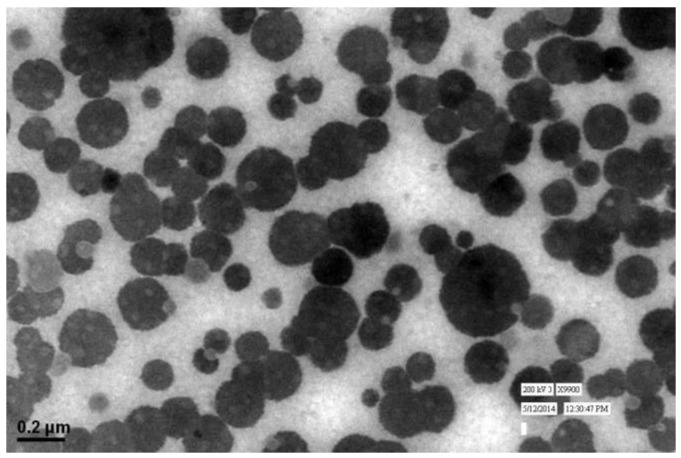
TEM image of NAR-HA@CH-PCL-NP.

The particle size and LBL assembly by respective polymers evaluated in NAR-HA@CH-PCL-NP through zetasizer and respective zeta potential values were further investigated for the surface morphology by Transmission Electron Microscopy (TEM). TEM micrograph of NAR-HA@CH-PCL-NP presented spherical nanoparticles as depicted in Figure 4. The cumulative percent intensity as revealed in Figure 2b indicated a narrow range of particle size distribution.

### 3.3. Drug Loading and Entrapment Efficiency

The formulations were optimized by evaluating the drug loading capacity and entrapment efficiency in NAR-PCL-NP. The entrapment and loading efficiency of NAR-PCL-NP, NAR-CH-PCL-NP NAR-HA@CH-PCL-NP were found to be 75.8 ± 0.23% and 3.9 ± 0.11%, 72.6 ± 0.42% and 2.9 ± 0.006%, and 67.2 ± 0.31% and 3.76 ± 0.016%, respectively. The drug loading and entrapment efficiency of all formulations is recorded in Table 1. The gradual decrease in entrapment efficiency may be attributed to the fabrication steps that lead to drug loss.

### 3.4. In Vitro Release Studies

**Figure 5:**
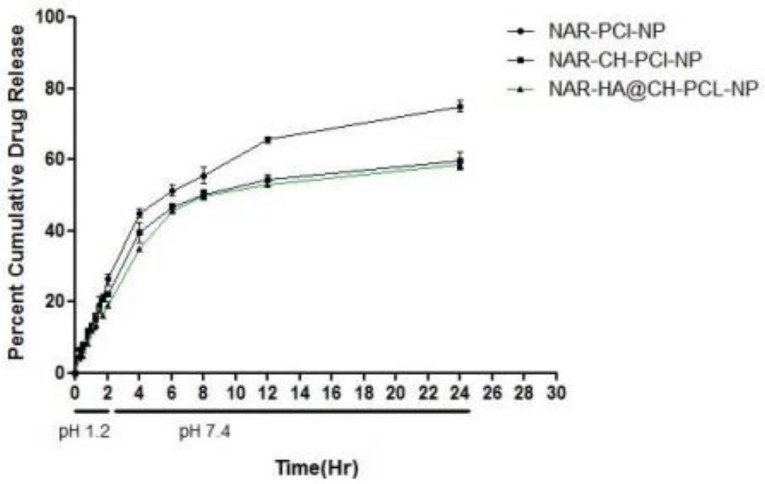
In Vitro release profile of NAR-PCL-NP, NAR-CH-PCL-NP and NAR-HA@CH-PCL-NP.

The release study was performed for 24 h, with initial 2 h in simulated gastric fluid (SGF) of pH 1.2 (2 h) and simulated intestinal fluid (SIF) of pH 7.4. The dissolution profiles of the unmodified and modified formulations are summarized in Figure 5. As the natural products are known to degrade in acidic medium, therefore the extent of drug released in acidic medium for oral delivery was also determined. Our results suggest that, the developed surface modified NPs are appropriate as oral delivery carriers and are capable of salvaging NAR from potential acid degradation, and facilitating its release in the intestine. The release of NAR-PCL-NPs, NAR-CH-PCL-NP and NAR-HA@CH-PCL-NP was 29%, 22% and 17% during the 2 h study. In pH 7.4, compared with NAR-CH- PCL-NPs, NAR-HA@CH- PCL NPs displayed a sustained and slower biphasic release profile. About 59% cumulative release of NAR from NAR-HA@CH-PCL-NP was observed in 24 h compared with 77% from NAR-PCL-NP. The release from unmodified NAR was significantly higher (*p* < 0.01) when compared with HA modified NAR. The possible reason behind the sustained release observed in case of NAR-HA@CH-PCL-NP could be the HA decorated CH coating over the PCL-NPs surface that caused increase in particle size, and it took a longer time for dissolution medium to penetrate through the polymer matrix into the interior of the NPs, thus leading to sustained and smooth drug release. The NAR-CH-PCL-NP and NAR-HA@CH-PCL-NP have similar release profile due to their surface modification with CH. It may be noted that the presence of targeting moiety would not have a significant effect on in vitro release.

### 3.5. Determination of Kinetic Model for Release of NAR-HA@CH-PCL-NP

Different kinetic models were applied to the data obtained from release study for categorizing the kinetics of drug release. In Table 2, it was observed and concluded that the Higuchi model gave the highest value of the squared correlation coefficient (R2), indicating that this mathematical model was most suitable for describing the release of the NAR from PCL nanoparticles. These results suggest that the release of NAR from PCL nanoparticles is controlled by diffusion [37]. From these results, the mechanism of the drug release from PCL nanoparticles can be considered to follow the sequence: (1) dissolution media penetrate into polymeric matrix of the nanoparticles through pores; (2) slowly dissolve the drug; and (3) NAR is released by diffusion in the acceptor solution.

### 3.6. Stability Studies

This study was designed to investigate the storage stability of the developed NAR-HA@CH-PCL-NP over a prolonged period. The NAR-HA@CH-PCL-NPs was stored under refrigeration (4 ± 1 °C) and ambient temperatures (25 ± 5 °C) and their stability was evaluated at pre-determined time-points during three-month study period. The samples were withdrawn at regular intervals and were analyzed for particle size, zeta potential and drug loading as the indicators of stability. In our experiment, we found that the particle size, zeta potential and drug loading had no significant effect on storage up to three months under both conditions, which means that our developed formulations were stable for at least three months. The data are presented in Table 3. Oral delivery of NPs faced a major hurdle when they encountered varying pH in GI tract. Therefore, stability study of NAR-HA@CH-PCL-NP was designed in acidic as well as basic pH values, including pH 2, pH 6.8, pH 7.4 and simulated GIT fluids. The size and zeta potential of NAR-HA@CH-PCL-NP were evaluated to investigate the pH-dependent stability. Table 4 displayed the stability profile of NAR-HA@CH-PCL-NP in varying test conditions. There was minor change in particle size of NAR-HA@CH-PCL-NP and also in significant shifts in PDI and zeta potential was observed ensuring the stability of NPs in varying pH conditions.

### 3.7. In Vitro Cell Studies

#### 3.7.1. Cell Cytotoxicity Study by MTT Assay

**Figure 6:**
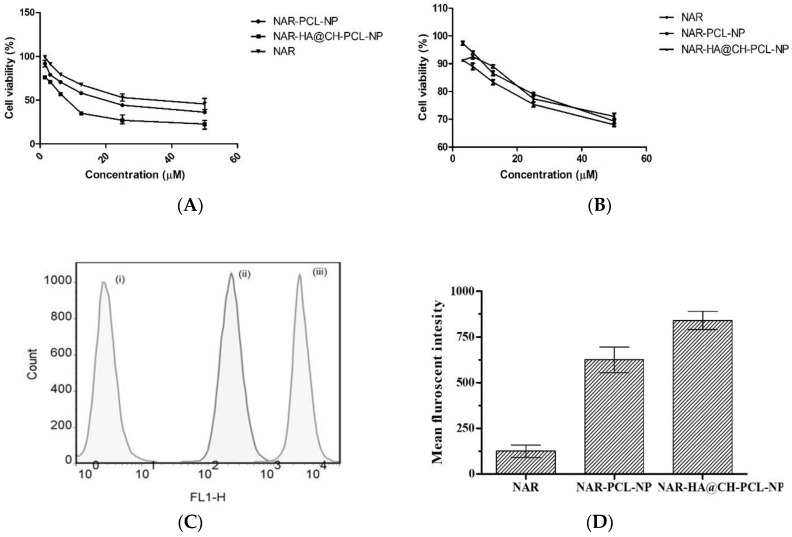
(**A**) Cytotoxic effect of various concentrations of NAR as pure NAR, NAR-PCL-NP and NAR-HA@CH-PCL-NP on A549 cells (**B**); safety evaluation of NAR, NAR-PCL-NP and NAR-HA@CH-PCL-NP on J774 macrophage cells (**C**); representation of uptake of NAR in A549 cells; and (**D**) bar graph representation of Cell uptake study: (i) Control; (ii) NAR-PCL-NP; and (iii) NAR-HA@CH-PCL-NP.

NAR-HA@CH-PCL-NP were evaluated for cytotoxicity on A549 lung cancer cells and J774 macrophage cells by MTT assay. The NAR, NAR-PCL-NP and NAR-HA@CH-PCL-NP were incubated with lung cancer cells and macrophage cells in the concentration range of 50–1.5 µM for 48 h. The results are represented in Figure 6a, and demonstrate a significant decrease in the IC_50_ of NAR-HA@CH-PCL-NP (5.33 µMl) in comparison to NAR-PCL-NP (11.5 µM) and NAR (25.1 µM) alone (Figure 6a,b).

#### 3.7.2. In Vitro Uptake Study in A549 Cells

In vitro cellular uptake study illustrated that the cell internalization of NAR in A549 cells is profoundly enhanced when formulated as NAR-HA@CH-PCL-NP (Figure 6c,d). NAR-HA@CH-PCL-NP, delivered 1.5 times higher payload of NAR than NAR-PCL-NP. Enhanced cellular uptake can be interconnected with depressed IC_50_ of NAR-HA@CH-PCL-NP. The uptake study served as relevant proof for functionalization of HA modified nanoparticles.

#### 3.7.3. Cell Cycle Arrest Assay

**Figure 7:**
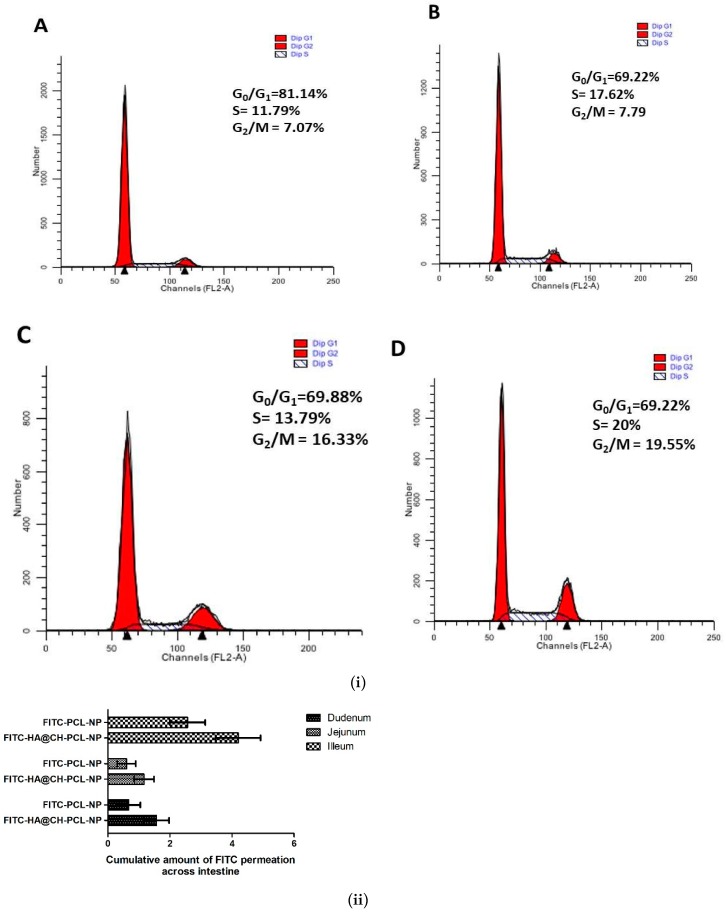
(**i**) Representative cell cycle distribution in A549 cells after treatment with: (**A**) control; (**B**) blank HA@CH-PCL-NP; (**C**) NAR; and (**D**) NAR-HA@CH-PCL-NP, at sub IC_50_ drug concentration. Analysis was performed using flow cytometry. Each figure individually represents the population present in G1-S and G2-M phases. (*n* = 3) (*p* < 0.05). (**ii**) Ex vivo permeation study of FITC loaded nanoparticles through rat intestine.

Cell cycle arrest by NAR and NAR-HA@CH-PCL-NP was investigated in A549 cells. NAR is known to inhibit MMP-2 and -9 as well as reduce the AKT activity in A549 cells, thereby causing cancer cell apoptosis. In our findings, a marked arrest was found in cancer cells in G2-M phase subsequent to apoptosis. Figure 7 displayed data of cell cycle study in A549 cells after incubation with free NAR, NAR-PCL-NP and NAR@HA-CH-PCL-NP. Treatment with free NAR and NAR-HA@CH-PCL-NP triggered cell cycle arrest in G2-M phase, whereas blank HA@CH-PCL-NP showed no significant change in G2-M phase. NAR-HA@CH-PCL-NP arrested the G2-M phase to a higher extent than NAR, gradual elevation in G2-M population to 19.55% after 24 h indicating limited cell growth restriction and cell division.

### 3.8. In Vivo Studies

#### 3.8.1. Ex Vivo FITC-Loaded HA Nanoparticles across Excised Rat Small Intestine

To investigate the HA presentation in encouraging the drug absorption, an ex vivo model was designed. The permeability of FITC-loaded HA nanoparticles through rat small intestine enclosing mucosa was determined ex vivo, and compared with FITC-PCL-NPs in Figure 7ii. To access the transport of FITC through intestine, small intestine was segmented into three sections: duodenum, jejunum and ileum and time-dependent study for FITC permeation was conducted. The results displayed in Figure 7ii gave an insight of transport by HA modified nanocarrier. The extent of permeation follows the order: ileum > duodenum > jejunum. Bar graph indicated that the amount of FITC that permeated across the ileum and duodenum were comparatively high in HA@CH-PCL-NP. Thereby, the study substantiates that HA modified nanoparticles contributed to drug transport through excised mucosa of rat small intestine.

#### 3.8.2. Tumor Regression Study

**Figure 8:**
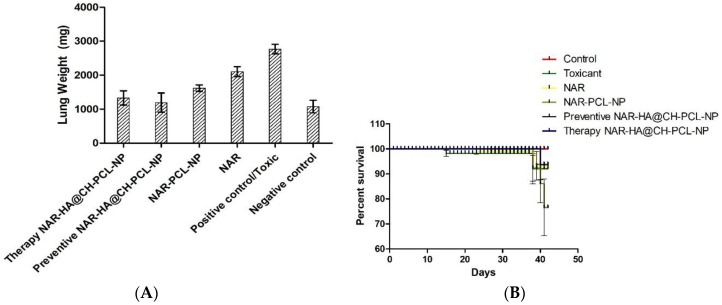

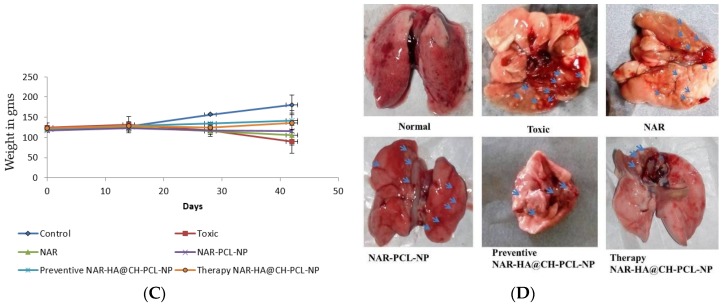
(**A**) Bar graph of lung weight of isolated lungs from urethane-induced SD rat tumor model after treatment with NAR, NAR-PCL, preventive NAR-HA@CH-PCL-NP (dosing initiated 15 days prior the tumor induction) and therapy NAR-HA@CH-PCL-NP (dosing start after induction of tumor); (**B**) survival graph with percent test animal survived versus time; (**C**) weight variation in animals during study; and (**D**) morphology of tumors in the harvested lungs at the end of the study.

The preventive and therapeutic effect of NAR-HA@CH-PCL-NP was compared with NAR and NAR-PCL-NP in in vivo urethane-induced tumor model. In preventive group, the dose was started 15 days prior to the induction of tumor as a result of carcinogen exposure. At the end point of study i.e., 21 days for Control, NAR, NAR-PCL-NP and therapy NAR-HA@CH-PCL-NP and 35 days for preventive NAR-HA@CH-PCL-NP after the start of treatment, the rats were sacrificed and the lungs were isolated. The lung weight was recorded and tumors were localized in the organ. Both preventive and therapeutic NAR-HA@CH-PCL-NP group displayed major regression in tumor marked by comparative low lung weight, whereas NAR-PCL-NP displayed minor regression in tumor at the end point of experiment (Figure 8a–d). The results revealed a significant enhancement of therapeutic efficacy (*p* < 0.1) when compared to toxic group. Survival plot depicted a significant reduction in animal death in both therapeutic and preventive therapy through NAR-HA@CH-PCL-NP when compared to toxic group (*p* < 0.05). Weight variations plots for test animals during study duration showed depressed weight reduction in test animals on therapeutic and preventive therapy NAR-HA@CH-PCL-NP as compared with toxicant, NAR and NAR-PCL-NP groups. The effect is correlated to the antioxidant potential of NAR for preventive activity and reported mechanism of reducing MMP-2 and -9 in lung cancer cells to inhibit cell proliferation.

#### 3.8.3. Histological Evaluation of Tumors

**Figure 9:**
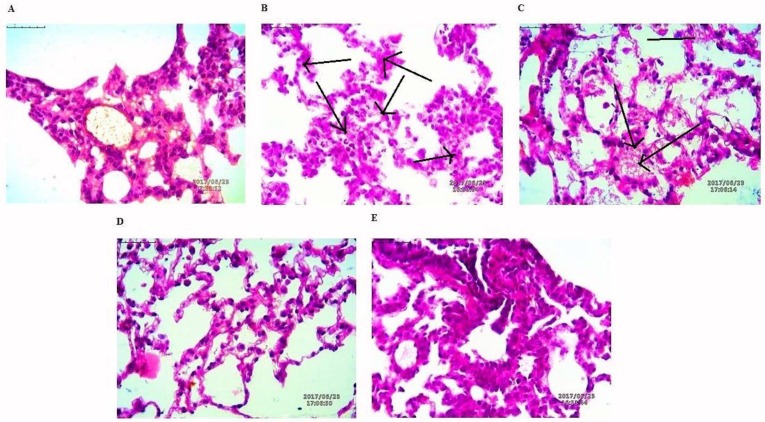
H-E staining micrographs of: (**A**) control; (**B**) toxicant; (**C**) NAR; (**D**) preventive NAR-HA@CH-PCL-NP; and (**E**) therapy NAR-HA@CH-PCL-NP. Black arrows indicate tumor stroma cells.

The lung tissues were histopathologically observed for their histological changes, as shown in Figure 9. A predominant solid pattern was present in H-E stained micrographs. Histological changes at the end of study toxic group high explicit tumor stroma cells represented by black arrows. In the lung tissues of rat treated with NAR-HA@CH-PCL-NP for 15 days prior to the toxicant, the structural integrity of the lung was less affected in the preventive group followed by the therapy group.

### 3.9. Biochemical Estimation

The effect of pure NAR, NAR-PCL-NP and NAR-HA@CH-PCL-NP was investigated on oxidative stress markers, i.e., TBARs, SOD, Catalase and Protein Carbonyl, in lung homogenate. The tumor bearing lungs were isolated from albino wistar rats challenged by intraperitoneal injection of urethane and were homogenized for further oxidative stress study. The TBARs and SOD levels significantly increased with urethane administration and were markedly decreased as a result of NAR-HA@CH-PCL-NP administration. The results reflect that NAR-HA@CH-PCL-NP also significantly (*p* < 0.5) restores the tissue catalase level that had previously diminished after urethane administration. In cancerous tissue, the protein level increased above normal and the same pattern was visible in urethane treated group. This increased protein carbonyl level is significantly decreased (*p* < 0.5) by NAR-HA@CH-PCL-NP to an almost normal level. Therefore, NAR-HA@CH-PCL-NP expressed an overall, superimposed antioxidant effect to reinstate the redox balance in preventive NAR-HA@CH-PCL-NP group in comparisons to therapy through NAR-HA@CH-PCL-NP, NAR-PCL-NP and pure NAR and toxicant group as shown in Table 5.

## 4. Discussion

Safe chemotherapy is an urgent requirement for management of cancer and elimination of associated toxicity of chemotherapy. Addressing this issue, several attempts have been made for development of effective delivery systems for natural anticancer agents [38]. Designing a drug delivery system for these natural agents is a challenging task [39,40]. In the present study, anticancer output of NAR has been investigated for repressing lung carcinoma. NAR is a potent flavonoid yet has limited investigation in in vitro and in vivo models for anti-cancerous activity due to less aqueous solubility and stability, low bioavailability and extensive first-pass metabolism. The development of polymeric nanoparticles deal with various parameters, including polymer type, surface modification, formulation technique, particle size and surface charge of NPs and the inherent features of the drugs [41,42]. PCL is a hydrophobic, biocompatible and biodegradable polymer which is successfully used to formulate nano-drug delivery systems. PCL has a fast releasing profile, thus various surface modifications with biodegradable polymers were attempted with PCL to improve its delivery aspects. The objective of these studies is to widen the range of functionality of PCL nano-particle release systems [42,43,44]. Thus, incorporation of CH surface coating controls the release profile of NPs and overcomes the limitations of PCL delivery [36].

Factorial design study contributes to the selection of the appropriate outputs for respective variables to design a most suitable nanocarrier system with PCL. In addition to theoretical outputs, the actual outputs of the formulations F1 to F8 were also correlated and zeta potential was also found to contribute to the intra-cellular localization of the NPs. The developed PCL NPs were characterized for their size, charge based stability, percent drug loading and entrapment efficiency [45]. The particle size of NAR-CH-PCL-NP and NAR-HA@CH-NP nanoparticles was found to be 242.8 ± 0.72 nm and 250 ± 1.1 nm respectively which was found to be in the desirable range for targeted delivery. On surface modification by CH coating, the multilayer coat results in an increase in the particle size of the nanoparticles. Therefore, NAR-HA@CH-NP has increased size as compared to NAR-CH-PCL-NP, NAR-CH-PCL-NP and NAR-HA@CH-NP have zeta potential 10.8 ± 0.012 and −24.24 ± 0.015 mV whereas in CH coated NAR-CH-PCL-NP the zeta potential shifts towards the positive side with increased zeta potential from −2.52 ± 0.015 to 10.8 ± 0.012 mV. The net positive zeta potential is due to CH surface modification whereas overlaid by HA the effective charge of NAR-CH-PCL-NP shifts towards the negative side and is 24.24 ± 0.015 which suggests that the method selected for development and optimization produced a stable nanoformulations [46,47]. Moreover, it is well reported that surface modification with hydrophilic polymers of NPs results in improved cell uptake and allows them to escape the opsonization process irrespective of their particle size [48,49]. This could be due to repeated circulation and prevention of RES uptake, which enables nanoparticle to be taken up by cancer cells owing to overexpression of CD44. In addition, CH coating rescues the therapeutic agents from acidic degradation in the stomach as CH is stable in acidic medium [33]. This also influences the % entrapment efficiency (EE) of delivery systems as revealed from the results. CH coated NAR-CH-PCL-NP have higher % EE of 72.6 ± 0.42% as compared to NAR-HA@CH-PCL-NP (67.2 ± 0.31%). The effect of CH coating was also observed in release profile of these nanoparticles, as the presence of CH surface coating on polymeric nanoparticles diminishes the release rate of NAR compared to the corresponding NAR-PCL-NP [50]. This could be attributed to the availability of drug only after the erosion of polymer, layer by layer as explained by Higuchi model [51].The formulation was quite stable in gastric pH during peroral administration retaining HA integrity as reported in the previous literature which could be due to the protection offered via formulation of NPs [52,53]. This could be ascribed to the proper coating of CH which extends the drug release in a sustained manner. HA, has a pKa value of 3.0 and possesses weak acidic nature, which results in lesser ionization of HA chains with a change in pH. The slow release rate of NAR from HA nanoparticles could be due to a very small degree of ionization of HA at pH 1.2, hence preventing it from strong-acidic gastric environment and delivering it an undamaged form to the small intestine [54]. The mechanism of protection against enzymatic or strong-acidic gastric environment could be attributed to HA coating which prevents nanoparticles from breaking down in the GI tract. These unbroken nanocarriers infiltrate into blood circulation and into tumors specifically because of enhanced permeability and retention (EPR) effect and the specific binding to CD44-receptor. Furthermore, the hyaluronidase-1 (Hyal-1) is extensively distributed in the acidic tumor extracellular matrix, which degrades HA coating and the nanoparticles deliver the drug into tissues and cells [55].The stability studies of NAR-HA@CH-PCL-NP were carried out for three months. The formulation was stable in this duration with respect to particle size, zeta potential and loading capacity due to the HA and CH coat over the PCL [33]. However, the stability of a colloidal system is more complex; although ZP does provide indications on colloid stability based on electrostatic repulsive forces. [50]. Enhanced arrest at G2-M phase of NAR-HA@CH-PCL-NP, when compared to NAR and enhanced survival rate of the animals in the treated group when compared with toxic group could both be due to enhanced localization and penetration of the drug into the leaky vasculature of tumor tissue and also the higher affinity of HA modified nanoparticles to overexpressed CD44. Tumor regression analysis proves chemopreventive property of NAR when formulated with HA (NAR-HA@CH-PCL-NP) as the drug has property to reduce oxidative stress, which plays a major role in carcinogenesis. Cytotoxicity assay revealed a significant (*p* < 0.05) detraction in cell viability on A549 cell lines while proven safe on J774 macrophages indicating safety on normal cells. The higher cytotoxicity can be attributed to the fact that modified NPs enter the cells through HA receptor-mediated endocytosis resulting in higher accumulation within the cells. A significantly (*p* < 0.01) higher therapeutic efficacy of NAR-HA@CH-PCL-NP was observed when compared with plain NAR. This could be attributed to targeting potential, stability and enhanced uptake of NAR-HA@CH-PCL-NP to the tumor tissue. The targeting potential was well proven by ex vivo intestine permeation study. This significant transport through the duodenum and ileum and shielding against enzymatic degradation could be attributed to the entrapment and shielding of NAR in nanoparticles. The enhanced targeting to the tumor could be again a result of opsonization and interaction of HA with CD44 overexpression in cancer tissues. Histopathological and biochemical results also proved the supremacy of NAR-HA@CH-PCL-NP over other formulations which could be a result of the above-mentioned factors, which effectively limits its degradation before reaching the targeted site.

## 5. Conclusions

Surface decorated PCL nanoparticles containing NAR were successfully developed, which were further coated with HA for tumor targeting. The NAR-HA@CH-PCL-NP proved potential in treating NSCLC and displayed the capability to reach programmed target site while protecting the encapsulated drug against broad pH variability throughout GIT as revealed by ex vivo permeation study. Peroral administration of NAR-HA@CH-PCL-NP produced significant decrease in tumor development and biochemical species levels, in comparison to NAR and NAR-CH-PCL-NP. Auxiliary chemopreventive potential of NAR was assessed against development of urethane-induced NSCLC in rat. The results obtained were positive as a fall in tumor numbers was observed. We can conclude from the results, that the developed formulation has promising potential as a chemopreventive as well as therapeutic agent for treating Lung cancer. The developed nanoformulation can be further commercialized as a marketed formulation, as the methods used for the preparation are simple, practical as well as economical and allow industrial scale up.

## Figures and Tables

**Table 1:** Particle size, PDI, zeta potential, Drug loading and entrapment efficiency of PCL formulations (All values expressed are mean ± SD where *n* = 3).

S. No.	Formulation Code	Particle Size (nm)	Zeta Potential (mV)	Polydispersity Index (PDI)	% Drug Loading	% Entrapment Efficiency
1	F1	215.6 ± 2.9	12.43 ± 0.045	0.227 ± 0.0015	2.3 ± 0.02	67.9 ± 0.06
2	F2	222 ± 0.36	19.54 ± 0.015	0.077 ± 0.0011	3.9 ± 0.012	75.8 ± 0.23
3	F3	196 ± 1.05	19.79 ± 0.079	0.599 ± 0.16	2.7± 0.01	60.3 ± 0.09
4	F4	198.3 ± 1.15	0.589 ± 0.001	0.147 ± 0.006	2.5 ± 0.016	65.6 ± 0.13
5	F5	178.5 ± 0.46	−8.15 ± 0.026	0.465 ± 0.003	2.3 ± 0.015	71.2 ± 0.10
6	F6	196.8 ± 0.35	−2.15 ± 0.011	0.152 ± 0.07	2.9 ± 0.006	54.03 ± 0.16
7	F7	186.9 ± 0.53	−1.81 ± 0.015	0.888 ± 0.24	2.7 ± 0.012	55.4 ± 0.13
8	F8	191.3 ± 0.3	−2.52 ± 0.015	0.238 ± 0.007	2.2 ± 0.01	57.8 ± 0.05
9	NAR-CH-PCL-NP	242.8 ± 0.72	10.8 ± 0.012	0.244 ± 0.003	2.9 ± 0.006	72.6 ± 0.42
10	NAR-HA@CH-PCL-NP	250.1 ± 1.1	−24.24 ± 0.015	0.263 ± 0.001	3.76 ± 0.016	67.2 ± 0.31

**Table 2:** Release kinetics for dissolution data of NAR-HA@CH-PCL-NP.

Formulation Code	Correlation Co-Efficient *R*^2^ Value			
	Zero Order	First Order	Higuchi Model	Korsmeyer Peppas Model
F2 (NAR-HA@CH-PCL-NP)	0.742	0.476	0.938	0.904

**Table 3:** Stability study data (in respect of particle size, zeta potential and percent drug loading) of NAR-HA@CH-PCL-NP for 3 months at 25 °C ± 2 °C and 4 °C ± 1 °C.

Sr. No.	Sampling Interval (Days)	Particle Size (nm)		Zeta Potential (mV)		% Drug Loading	
		25 °C ± 5 °C	4 °C ± 1 °C	25 °C ± 5 °C	4 °C ± 1 °C	25 °C ± 5 °C	4° C ± 1 °C
1	0	251.2 ± 2.67	251.2 ± 1.44	−24.25 ± 0.05	−24.25 ± 0.4	3.76 ± 0.03	3.76 ± 0.03
2	30	249.5 ± 2.11	250.9 ± 2.11	−23.76 ± 1.05	−23.9 ± 0.34	3.62 ± 0.02	3.68 ± 0.12
3	60	253.6 ± 3.02	252.1 ± 3.43	−22.8 ± 0.04	−23.4 ± 0.12	3.5 ± 0.01	3.57 ± 0.31
4	90	263 ± 1.90	255 ± 1.36	−22.6 ± 0.17	−23.2 ± 0.51	3.45 ± 0.01	3.5 ± 0.06

**Table 4:** Stability profile of NAR-HA@CH-PCL-NP after exposure to different pH conditions.

pH Condition	Particle Size (nm)	PDI	Zeta Potential (mV)
Initial	251.2 ± 12.3	0.263 ± 0.001	−24.25 ± 0.015
pH 2 ^†^	211.6 ± 11.2	0.223 ± 0.013	−20.5 ± 0.64
pH 6.8 ^††^	228.1 ± 8.7	0.113 ± 0.006	−22.2 ± 0.71
pH 7.4 ^†††^	257.2 ± 15.4	0.119 ± 0.008	−24.3 ± 0.02
SGF ^†^	230.3 ± 12.2	0.213 ± 0.001	−21.2 ± 0.33
SIF ^†††^	245.3 ± 14.2	0.344 ± 0.011	−25.3 ± 0.51
Culture media ^††††^	255.6 ± 19.5	0.256 ± 0.003	−24.8 ± 0.44

^†^ Incubation for 2 h. ^††^ Incubation for 6 h. ^†††^ Incubation for 24 h. ^††††^ Incubation for 7 days.

**Table 5:** Effect of pure NAR, unmodified NPs and modified NPs on oxidative stress markers, from lung homogenate in Urethane-induced lung cancer. (Significant values (* *p* < 0.05. ** *p* < 0.01, *** *p* < 0.001)).

Groups	TBARs (nm of MDA/µg of Protein	SOD (Units of SOD/mg of Protein)	CATALASE (nm of H_2_O_2_/min/mg of Protein)	PROTEIN CARBONYL *n* mol/mg of Protein
Control (Blank formulation oral)	191.51 ± 10.2 ***	0.0347 ± 0.003 *	0.402 ± 0.0183 ***	15.35 ± 0.08
Toxicant (urethane 1 g/kg i.p)	509.81 ± 16.14	0.043 ± 0.0056	0.115424 ± 0.13	41.56 ± 46.75
Urethane 1 g/kg + preventive NAR-HA@CH-PCL-NP 50 mg/kg oral	445.4 ± 5.21	0.0342 ± 0.0026 **	0.26 ± 0.012	30.4 ± 13.8
Urethane 1 g/kg + Therapy NAR-HA@CH-PCL-NP 50 mg/kg oral	128.21 ± 149.13 ***	0.025 ± 0.003 ***	0.380 ± 0.057 ***	21.64 ± 0.37
Urethane 1 g/kg + Pure NAR 50 mg/kg oral	271.9 ± 25.8 ***	0.03 ± 0.0053 ***	0.112 ± 0.12	33.05 ± 16.8

## Supplementary Materials

The following are available online at http://www.mdpi.com/1999-4923/10/1/33/s1. Table S1. Factors and their levels with codes for PCL formulations; Table S2. 23 full factorial design layouts for optimization of PCL formulations.

Supplementary File 1
