# Supplementary material for: Hyaluronic Acid Decorated Naringenin Nanoparticles: Appraisal of Chemopreventive and Curative Potential for Lung Cancer
Source: Pharmaceutics. 2018 Mar 12;10(1):33. doi: 10.3390/pharmaceutics10010033 (PMC5874846; doi:10.3390/pharmaceutics10010033)
Supplement: Supplementary File 1 [file pharmaceutics-10-00033-s001.pdf]

Hyaluronic Acid Decorated Naringenin Nanoparticles: Appraisal of Chemopreventive and Curative Potential for Lung Cancer

Poonam Parashar, Meena Rathor, Monika Dwivedi, Shubhini A Saraf*

Table S1. Factors and their levels with codes for PCL formulations.

Variables	Levels	
	Low (−1)	High (+1)
A (amount of PCL in %w/v)	0.5	1
B (amount of Tween 80 in %w/v)	0.5	1
C (Stirring speed in rpm)	600	800

Table S2. 2^3 full factorial design layouts for optimization of PCL formulations.

Formulation code	Factor 1	Factor 2	Factor 3
F1	+1	+1	−1
F2	+1	−1	−1
F3	−1	+1	+1
F4	+1	+1	+1
F5	−1	−1	+1
F6	+1	−1	+1
F7	−1	+1	−1
F8	−1	−1	−1
